# Draft Genome Sequences of Two Natural Isolates of the Yeast Barnettozyma californica from Ireland, UCD09 and UCD89

**DOI:** 10.1128/genomeA.00548-18

**Published:** 2018-06-21

**Authors:** Alisha A. Mullen, Ciara D. Lynch, Shannon M. Hill, Cian P. Holohan, Tadhg Ó Cróinín, Kenneth H. Wolfe, Caoimhe E. O’Brien, Geraldine Butler

**Affiliations:** a School of Biomedical and Biomolecular Sciences, Conway Institute, University College Dublin, Dublin, Ireland; b School of Medicine, Conway Institute, University College Dublin, Dublin, Ireland

## Abstract

No genome sequence of a species from Barnettozyma, a yeast genus in the family Phaffomycetaceae, is currently available. We isolated two B. californica strains from soils in Ireland and generated draft sequences of their 11.7-Mb genomes. Single nucleotide polymorphism (SNP) analysis showed 20,490 differences between the strains and suggests that B. californica is haploid.

## GENOME ANNOUNCEMENT

We isolated yeasts from soil samples from Ireland following the protocol of Sylvester et al. (1). Samples were incubated at room temperature in yeast extract-peptone-dextrose (YPD) broth containing carbenicillin (10% [wt/vol]) and chloramphenicol (3% [wt/vol]). Following isolation of single colonies, species were identified by sequencing the internal transcribed spacer (ITS) region of rDNA. Two isolates, UCD09 and UCD89, were identified as Barnettozyma californica by searches against the YeastIP database (2). UCD09 was isolated from a wooded area at the Special Ops Paintball Range in Roundwood, County Wicklow, Ireland, and UCD89 from a patch of grass at Balally Park, Dublin, Ireland, about 25 km apart.

B. californica has a worldwide distribution and has previously been isolated from substrates including soil, water, animal dung, tree fluxes, and rotting wood (3, 4). The type strain, CBS 252, was isolated from soil in California in 1931 (5). B. californica has been found on the surfaces of olives and secretes lipase in sufficient amounts that it can degrade the quality of olive oil in storage (6). It is one of the six known species in the genus Barnettozyma and was formerly called Williopsis californica (3, 7). No genome sequence has previously been reported for any species in this genus. Barnettozyma is in the family Phaffomycetaceae, which also includes the genera Wickerhamomyces, Cyberlindnera, Starmera, and Phaffomyces (8).

Genomic DNAs were isolated by phenol-chloroform extraction and cleanup (DNA Clean & Concentrator-25 kit, Zymo Research). Sequencing was done by BGI Tech Solutions using an Illumina HiSeq 4000 instrument. Libraries with 170- to 800-bp inserts were generated, and 150 bases were sequenced from each end. Low-quality reads were removed using Skewer (9). The reads were then assembled into scaffolds using SPAdes version 3.11.1 (10). To remove contaminants, scaffolds with coverages below 10× or lengths below 1 kb were discarded. The final sets of scaffolds were quality analyzed using Quality Assessment Tool for Genome Assemblies (QUAST) (11).

The UCD09 assembly totaled 11.67 Mb, the *N*_50_ value was 164 kb, the longest scaffold was 415 kb, average coverage was 76×, and there were 164 contigs of >1 kb. For UCD89, the assembly totaled 11.68 Mb, the *N*_50_ value was 169 kb, the longest scaffold was 757 kb, average coverage was 69×, and there were 169 contigs of >1 kb. Protein-coding genes were annotated using AUGUSTUS (12) version 2.5.5 (with a Saccharomyces cerevisiae training set), which predicted 5,803 genes in UCD09 and 5,802 genes in UCD89. tRNAscan-SE version 1.3.1 (13) predicted 200 (UCD09) and 199 (UCD89) tRNA genes.

Single nucleotide polymorphisms were identified using the Burrows-Wheeler Aligner (BWA) (14) and SAMtools (15). The numbers of SNPs within each strain were low (1,482 in UCD09; 1,714 in UCD89), and the distribution of allele frequencies at SNP sites was flat, suggesting that both strains are haploid. Mapping the reads from UCD09 to the UCD89 assembly identified 20,490 SNPs between these two natural isolates. For comparison, strains of Saccharomyces paradoxus from Europe typically differ by 10,000 SNPs (16).

### Accession number(s).

These whole-genome shotgun projects have been deposited in DDBJ/ENA/GenBank under the accession no. QBLI00000000 and QBLJ00000000. The versions described in this paper are the first versions, QBLI01000000 and QBLJ01000000.
